# ﻿*Anoectochiluszhongshanensis* (Orchidaceae), a new species from Guangxi, China

**DOI:** 10.3897/phytokeys.234.111106

**Published:** 2023-10-25

**Authors:** Yan-Bin Wu, Yu Han, Xu-Hui He, Hui-Ling Chen, Jin-Zhong Wu, Qi Ye, Cheng-Jian Zheng

**Affiliations:** 1 School of Pharmacy, Fujian University of Traditional Chinese Medicine, 1 Qiuyang Road, Fuzhou 350122, China Fujian University of Traditional Chinese Medicine Fuzhou China; 2 Department of Chinese Medicine Authentication, Faculty of Pharmacy, Naval Medical University, 325 Guohe Road, Shanghai 200433, China Naval Medical University Shanghai China; 3 College of Life Sciences, Fujian Agriculture and Forestry University, Fuzhou 350001, China Fujian Agriculture and Forestry University Fuzhou China; 4 State Key Laboratory for Quality Ensurance and Sustainable Use of Dao-di Herbs, Beijng 100700, China State Key Laboratory for Quality Ensurance and Sustainable Use of Dao-di Herbs Beijng China

**Keywords:** *Anoectochilus*, new species, phylogeny, taxonomy

## Abstract

A new species of *Anoectochilus* (Orchidaceae) from Guangxi, China, *A.zhongshanensis*, is described here, which was identified based on phylogenetic studies adopting combined plastid markers (*rbcL*-*matK*-*trnL*-*F*), morphological observation and chemical analysis. Molecular phylogenetic results support the systematic status of *A.zhongshanensis* as a new species in *Anoectochilus* genus. Morphologically, this new species is similar to *A.zhejiangensis* and *A.malipoensis*, but differs by its characteristic labellum and column, including the hastate or scalpel-shaped lobes of epichile, forward curved and pinnately divided cristate lobes at both sides of the mesochile and inverted triangle column wings. Furthermore, HPLC-ELSD analysis of these three species revealed the interesting chemotaxonomic difference that the principle and characteristic lactone glycoside in this new species was kinsenoside, rather than its diastereoisomer, goodyeroside A, a major glycoside in *A.zhejiangensis* and *A.malipoensis*.

## ﻿Introduction

The genus *Anoectochilus* Blume (Goodyerinae, Cranichideae, Orchidaceae) consists of about 40 reported species in the world, distributed mainly from south and southeast Asia to Australia and the southwest Pacific islands (Jin et al. 2021; POWO 2023). To date, a total of 20 species (including 11 endemics) have been recorded from China (Tian and Xing 2008; Tian et al. 2014; Jin et al. 2021; POWO 2023). These *Anoectochilus* species, along with certain members of *Goodyera* and some related genera, are famous as Jewel Orchids in English (Cavestro 1994), and known as “Jin Xian Lian” in Chinese for their “golden lines” on leaves, with both medicinal and edible values.

In the course of our comprehensive resource survey of *Anoectochilus* species in China from 2015 to 2021, we have reported three new record species: *A.elatus* (Zheng et al. 2018), *A.papillosus* (Zheng et al. 2019) and *A.brevilabris* (Wu et al. 2017), mainly based on their plant morphology. Chemical diversity could also provide some taxonomic evidence for the quality control and authentication of *Anoectochilus* species using kinsenoside and goodyeroside A (a pair of diastereoisomers) as promising chemotaxonomic markers (Wu et al. 2020). However, species delimitation in this genus is still a big challenge and interspecific relationships among *Anoectochilus* species remain unclear. Molecular markers have therefore been more and more explored and utilized to solve those phylogenetic issues. ITS2 region as DNA barcode has been commonly adopted for distinguishing those related species, and also for the rapid discrimination of *A.roxburghii* and its counterfeits (Lv et al. 2015). Our recent study also indicated that both ITS2 and *psbA*-*trnH* sequences can be used to distinguish *A.roxburghii* from its related species with larger genetic distances (Wu et al. 2022b). By analyzing 20 species containing 58 samples, Han et al found that the combination of chloroplast gene fragments (*rbcL*-*matK*-*trnL*-*F*) was more helpful in exploring the infrageneric relationships among *Anoectochilus* species than any of those three individual DNA sequences (Han 2019). A similar strategy has been more recently adopted to identify a new *Anoectochilus* species, *A.medogensis*, from Tibet, China (Jin et al. 2021).

During our plant resource investigation in Zhongshan, Guangxi Province, China in Aug 2020, an *Anoectochilus* species was found to be difficult to identify, which was finally clarified as a new species on the basis of detailed morphological, molecular and chemical studies, and described below as *A.zhongshanensis* C.J. Zheng & Y.B. Wu. In this study, we deciphered the morphological differences between the new species and its nearest congener, *A.zhejiangensis* Z. Wei & Y. B. Chang. We also used three combined cpDNA sequences (*matK*, *trnL*-*F* and *rbcL*) to infer the phylogenetic relationships and substantiate the systematic status of *A.zhongshanensis* as a new species in *Anoectochilus*. In addition, potential chemotaxonomic markers, kinsenoside and goodyeroside A, were also employed to disclose the chemical difference between the new species and its nearest congeners, *A.zhejiangensis* and *A.malipoensis*.

## ﻿Materials and methods

Voucher specimens of *A.zhongshanensis* were collected in Zhongshan, Guangxi Province, and preserved at the herbarium of Fujian Agriculture and Forestry University (**FAFU**!). Fresh leaves were washed and dried with filter paper in the field, and then stored in a plastic bag with silica gel for molecular experiments. The living plants of *A.zhongshanensis* were carefully observed for detailed morphological description and local observation of the plant′s small parts was performed using a stereo microscope (SZ61). Tissue cultures of all collected *Anoectochilus* species in our lab have been successfully established for resource protection and further chemical and biological studies.

A total of 42 samples representing 18 *Anoectochilus* species were included for molecular analysis, and all sequences used for constructing the phylogenetic tree were downloaded from GenBank (Suppl. material 6) except those of the new species. *Zeuxinellavietnamica* was selected as anoutgroup according to previous phylogenic studies (Averyanov 1988; Averyanov and Averyanova 2003). To investigate the phylogenetic status of the new species within the genus *Anoectochilus*, four DNA markers, including internal transcribed spacer (ITS) and three plastid DNA regions (*matK*, *rbcL* and *trnL*-*F*), were selected to reconstruct the phylogenetic tree based on previous studies (Jin et al. 2021; Tong et al. 2022).

Ezup column plant tissue genomic DNA extraction kit (Sangon B518261) was used to extract the total genomic DNA from silica gel-dried leaves of the new species. The concentration of DNA samples used in this study were ≥ 20 ng/µL, and the working DNA was stocked in refrigerator at 4 °C for use. Polymerase Chain Reaction (PCR) amplification was performed on a Veriti 96-well thermal cycler (Verity, ABI, USA) using a 25 μL reaction system containing 2.5 μL 10× Taq Buffer (with MgCl_2_), 0.2 μL Taq enzyme, 1.0 μL Dntp (mix), 1.0 μL forward and reverse primers, 1.0 μL target DNA template and 18.3 μL ddH_2_O. Information on primers and amplification protocols for each DNA region is listed in Suppl. material 7.

DNA quality was detected by electrophoresis using 1% agarose and 1× TAE buffer solution (voltage 120–180V). The concentration and purity were detected by spectrophotometer, and gel imager FR-980A (Shanghai Furi Technology Co., LTD.) was used to record and take photos. The qualified PCR products were sequenced bi-directionally on a 3730XL sequencer (ABI, USA) after purified by a SanPrep column DNA gel extraction kit (Sangon B518131). Sequences were first assembled and edited with SEQMAN (DNA STAR package, USA), followed by sequence alignment with MEGA11 to trim the irregular bases at both ends of the aligned sequences. Bayesian inference (BI), maximum likelihood (ML) and maximum parsimony (MP) methods were used to construct the dataset of multi-gene tandem (*rbcL*-*matK*-*trnL*-*F*). All characters are considered as unordered and equally weighted, while the indels were processed as missing data after sequence alignment.

MP analysis was performed using MEGA11 (Felsenstein 1985; Tamura et al. 2021). Bootstrap values were generated with 1000 bootstrap replicates with the Subtree-Pruning-Regrafting (SPR) algorithm, with a search level of 1 in which the initial trees were obtained by the random addition of 10 sequences and a limit of 1000 trees, and branch lengths were calculated using the average pathway method. ML analysis of concatenated cpDNA by IQ-TREE-1.6.2 runs with aligned partitions and allows ModelFinder (Kalyaanamoorthy et al. 2017) to identify the best model for each partition (Table 1). Node support was estimated using 1,000 bootstrap iterations and other parameters were set as default for the searches. The BI analysis was performed using MRBAYES-3.2.7-WIN (Ronquist and Huelsenbeck 2003) with best-fit evolutionary models selected under the Bayesian Information Criterion (BIC) also using ModelFinder (Table 1), and the analysis consisted of 2,000,000 generations of four simultaneous Monte Carlo Markov chains. We increased the number of generations (typically 1,000,000) until the average standard deviation of split frequencies falls below 0.01 (3,000,000 generations in total). Phylogenetic trees were sampled every 1000 generations. When the average standard deviation of split frequencies consistently fails to reach the ideal values, the *run.1.p file can be viewed by Tracer v.1.6. The effective sample size (ESS) of all parameters were > 200. Posterior probability (PP) ≥ 0.95 or bootstrap values (BS_ML_, BS_MP_) ≥ 85 indicates strong support, whereas 0.95 > PP ≥ 0.85 or 85 > BS_ML_, BS_MP_ ≥ 70 suggests moderate support and weak support otherwise.

**Table 1.** nr/cpDNA data partitions and best-fit models estimated by IQ-TREE model selection for BI analysis.

Partition	Model
ITS	K80
*rbcL*	HKY
*matK*	GTR+I+G
*trnL*-*F*	HKY

HPLC-ELSD was performed using EasySep®-3030 HPLC system (Shanghai Tongwei Analytical Technology Co., LTD., China) equipped with an AQ-C18 chromatographic column (3 µm, 4.6 × 250 mm) and an ELSD detector. The mobile phase was ultrapure water (100%) and the flow rate was set at 0.5 mL/ min. The column temperature and ELSD spray chamber temperature were 30 °C and 70 °C, respectively, while the nitrogen flow rate was 2.5 mL/min (Wu et al. 2020). Tissue cultured plant samples of *A.zhongshanensis*, *A.zhejiangensis* and *A.malipoensis* were dried and ground into powder. 0.1 g powder of each sample was accurately weighed and ultrasonically extracted with 20 mL distilled water for 45 min. The extract was subsequently passed through a 0.22 µm PTFE syringe filter for HPLC-ELSD analysis.

## ﻿Results

**Figure 1.**
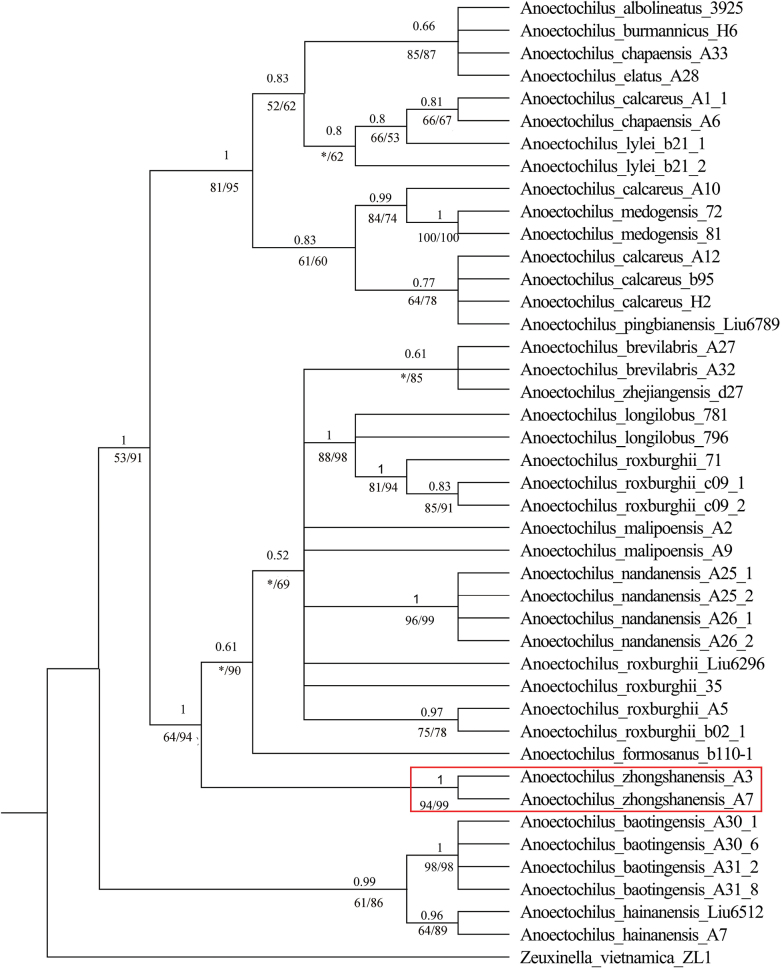
Phylogenetic relationships based on concatenated *rbcL*, *matK* and *trnL*-*F* sequences in *Anoectochilus* species inferred by Bayesian inference. PP_BI_ is shown above the branches, while BS_ML_ and BS_MP_ are displayed below the brancher (left, BS_MP_; right, BS_ML_). “*” indicates that the value is not supported or is smaller than 50.

Since ITS2 sequence alone could not confirm the systematic status of the new species with convincing infrageneric relationships in the tested *Anoectochilus* species (Suppl. materials 1–3), combined cpDNA sequences (*matK*, *trnL*-*F* and *rbcL*) were used to construct datasets that may provide more discriminative information in phylogenetic analysis. By comparing the phylogenetic trees of BI, MP and ML, it was found that the topologies of ML and BI analysis were almost the same, and the topology of MP tree was also congruent with those of ML and BI, only with slight differences in the largest clade including the new species (Suppl. material 4). Here, the topology diagram generated by BI is displayed as Fig. 1, with most of the major clades receiving relatively strong support (PP_BI_ ≥ 0.95). This BI phylogenetic tree revealed that two endemic species of Hainan Island, *A.hainanensis* and *A.baotingensis*, split off first and formed a branch as the sister clade of the other 16 species from mainland China with relatively strong support (PP = 0.99, BS_ML_ = 61, BS_MP_ = 86). The other 16 species separated into two large clades. Our two samples of *A.zhongshanensis* clustered into a well-supported subclade (PP_BI_ = 1, BS_MP_ = 94, BS_ML_ = 99), which is nested in the clade consisting of seven other *Anoectochilus* species, including *A.brevilabris*, *A.zhejiangensis*, *A.longilobus*, *A.roxburghii*, *A.malipoensis*, *A.nandanensis*, and *A.formosanus*. These phylogenetic analyses therefore supported the recognition of *A.zhongshanensis* as a new species of the genus *Anoectochilus*.

**Figure 2.**
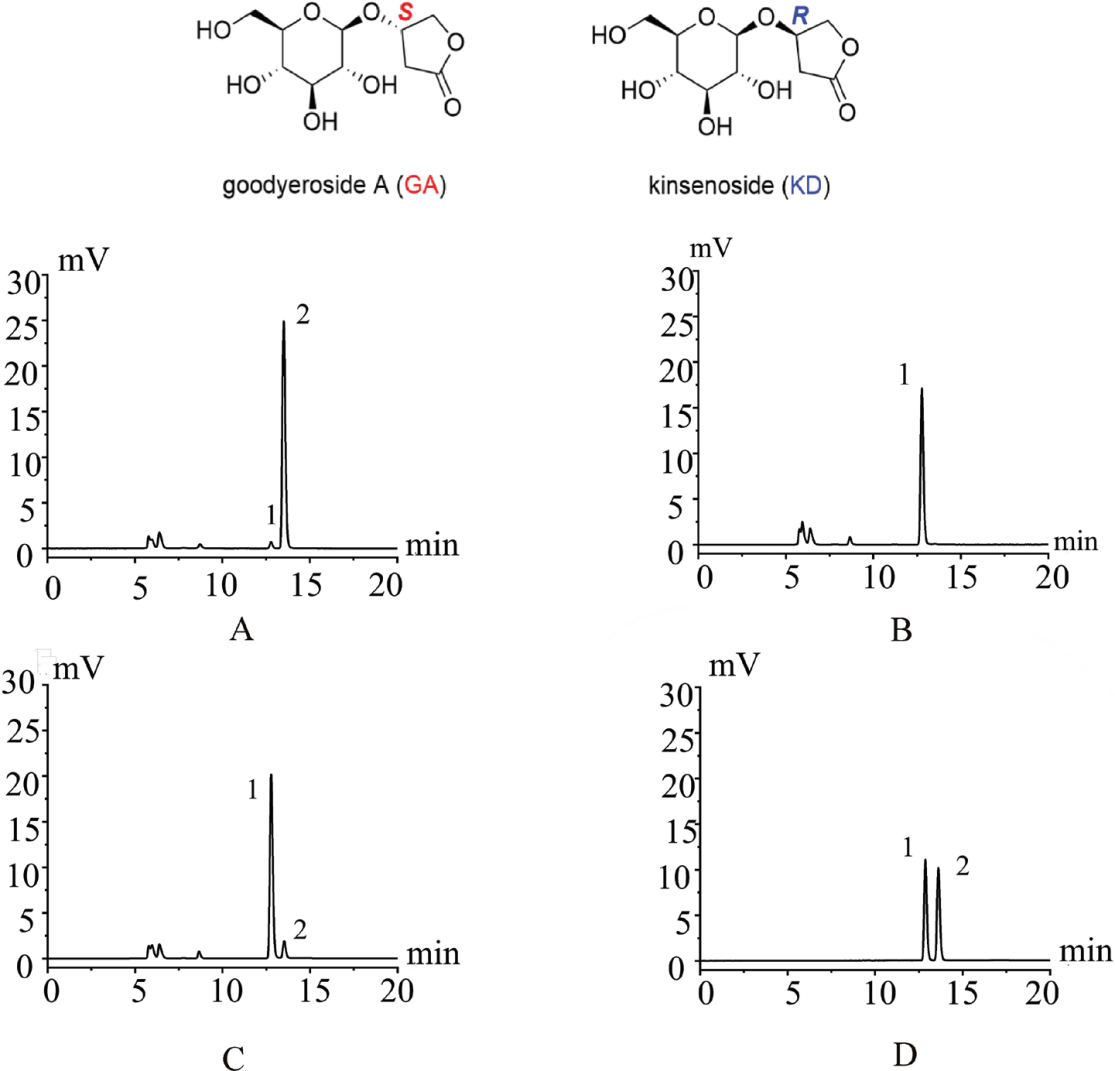
HPLC-ELSD chromatograms of *A.zhongshanensis* (**A**), *A.malipoensis* (**B**), *A.zhejiangensis* (**C**), and standards mixture (**D**) of goodyeroside A (1) and kinsenoside (2).

In addition, HPLC-ELSD analysis displayed interesting chemotaxonomic difference that the principle and characteristic lactone glycoside in *A.zhongshanensis* was kinsenoside rather than its diastereoisomer, goodyeroside A, a major glycoside in *A.zhejiangensis* and *A.malipoensis* (Fig. 2). Though morphologically similar, these three species are genetically and chemically different. Especially, those HPLC profiles revealed that *A.malipoensis* probably only contained goodyeroside A or with trace amounts of kinsenoside that cannot be detected, whereas the other two species have a mixture of goodyeroside A and kinsenoside but with inverse proportion.

### ﻿Taxonomic treatment

#### ﻿ Anoectochilus zhongshanensis

Taxon classificationPlantaeAsparagalesOrchidaceae

C.J.Zheng & Y.B.Wu sp. nov.

85CC1F3B-24AD-584F-8E54-17A58769C5AC

urn:lsid:ipni.org:names:77329347-1

Figs 3 , 7

##### Type.

China. Guangxi province: Zhongshan County, Hezhou City, under evergreen broad-leaved forest or shady and humid valleys, cultivated at the Medicinal Botanical Garden of the Second Military Medical University, 12 August 2020, Wu20200812003 (holotype: FAFU!).

##### Diagnosis.

*A.zhongshanensis* is similar to *A.zhejiangensis*, but can be distinguished by the hastate or scalpel-shaped lobes of epichile (vs. semiovoid), forward curved and pinnately divided cristate lobes at both sides of the mesochile (vs. backward curved, the same orientation as the spur), unbowed conical spur (vs. bowed) and inverted triangle column wings (vs. squarish) (Fig. 6). *A.zhongshanensis* is also similar to *A.malipoensis*, but can be distinguished by the hastate or scalpel-shaped lobes of epichile (vs. obovate lobes with acuminate apex and crenulate margins), pinnately divided cristate lobes at both sides of the mesochile (vs. obliquely subquadrate and serrate lobes) and inverted triangle column wings (vs. elliptic) (Chen and Shui 2010).

##### Description.

Terrestrial herb, 8~22 cm tall, with an erect stem and 2~6 leaves. Leaf ovate or orbicular, 1.2–4.0 × 1.0–2.8 cm, adaxially black with fine golden red net veins with silk luster, abaxially purplish red, apex acute, base subtruncate or rounded, abruptly narrowed into a stalk; petiole 4–12 mm long, base enlarged into a cauline sheath. Racemose inflorescence, 1–6 flowered, inflorescence rachis pubescent; peduncle long and slender, mauve red, pubescent, with 2–4 mauve sheath-like bracts; floral bracts reddish, ovate-lanceolate, ca. 6 × 3 mm, apex acuminate, abaxially pubescent, subequal length as the ovary or slightly longer; ovary cylindrical, not twisted, reddish brown, white pubescent, connected with pedicel ca. 13 mm long; flowers not resupinate (labellum held uppermost); sepals reddish, subequal, ca. 5 mm long, abaxially puberulent; dorsal sepal ovate, sunken navicular, apex acute, joined with petals to form a hood; lateral sepals oblong, slightly oblique; petals white greenish, obliquely falcate, ca.5 mm long, middle part ca. 1.5 mm wide, base narrow, apex acute; lip white, upstretched, Y-shaped, 13 mm long, epichile longitudinally dilated, 2-lobed, lobes hastate or scalpel-shaped, 5 mm long, ca. 2 mm wide, apex blunt, diverging at an acute angle; mesochile ca. 4 mm long, flange bearing forward curved and pinnately divided cristate lobes at both sides; conical spur, ca. 10 mm long, obliquely upward, subvertical to ovary, apex shallowly bilobed, containing 2 subcuneate calli. Column ca. 4 mm long, ventrally with an inverted triangular column wing on both sides. Anther cap ovate, ca. 3 mm. Pollinia 2, yellow, obliquely clavate, ca. 3 mm long. Rostellum erect, apical concave and bifurcated. Stigma lobes 2, distinct, located to the sides of the rostellum. Fruit not seen.

##### Etymology.

Referring to the locality (Zhongshan County) where this new species was found.

##### Vernacular name.

钟山金线兰 (Chinese pinyin: zhong shan jin xian lan).

##### Distribution and habitat.

*A.zhongshanensis* is currently known only from Zhongshan County, Hezhou City, Guangxi Province, China. This species grows in evergreen broad-leaved forests or shady and humid valleys, elev. 500–1200 m.

##### Conservation status.

During our three surveys in April, August and September 2020, *Anoectochiluszhongshanensis* was found in the forests or shady and humid valleys of Zhongshan County only at two separate locations, where we counted fewer than 100 individuals at each site. Due to the highly medicinal and edible value of *Anoectochilus* plants (Ye et al. 2017; Wu et al. 2022a; Shi et al. 2023), over exploitation and collection remains a high-risk factor for continuing resource decline and thus the major threat to these species, especially those with restricted distribution and small population size. All *Anoectochilus* plants have already been listed as potentially endangered species in the ‘List of National Key Protected Wild Plants in China’ (Level II) issued on Sep 7, 2021 by the National Forestry and Grassland Administration, and Ministry of Agriculture and Rural Affairs of the People’s Republic of China (http://www.gov.cn/zhengce/2021-09/07/content_5727413.htm). Though the comprehensive population assessment of *A.zhongshanensis* in the whole Zhongshan County has not been conducted, conservation status of this new species is best classified as ‘Endangered’ (En) (IUCN guidelines 2020) based on the discovered small population size of less than 200 mature individuals and potential risk of continuing decline in the number of mature individuals.

##### Phenology.

Flowering in August-October.

## ﻿Discussion

**Figure 3.**
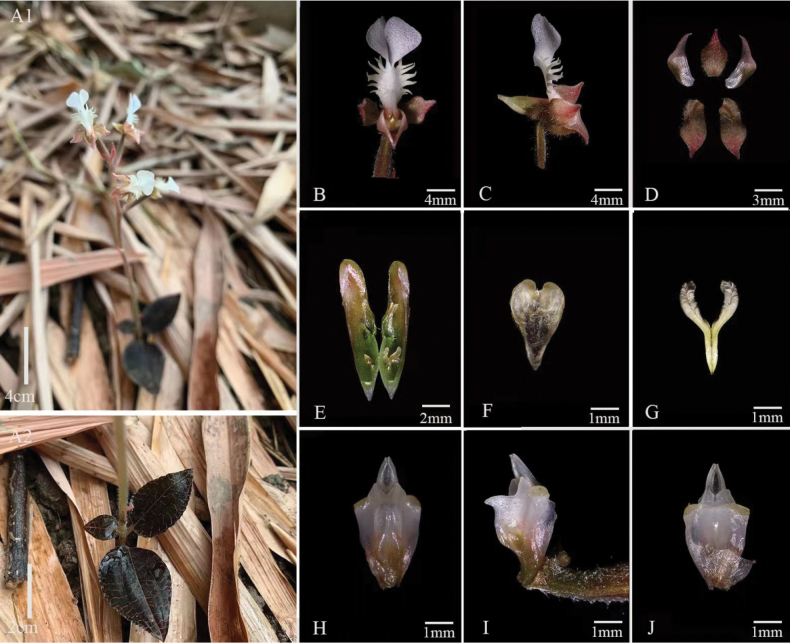
*Anoectochiluszhongshanensis***A1** and **A2** habit **B** flower (front view) **C** flower (lateral view) **D** sepals and petals **E** spur **F** anther cap **G** pollinia **H** column (front view) **I** column (lateral view) **J** column (rear view). Photographs by Yan-Bin Wu.

**Figure 4.**
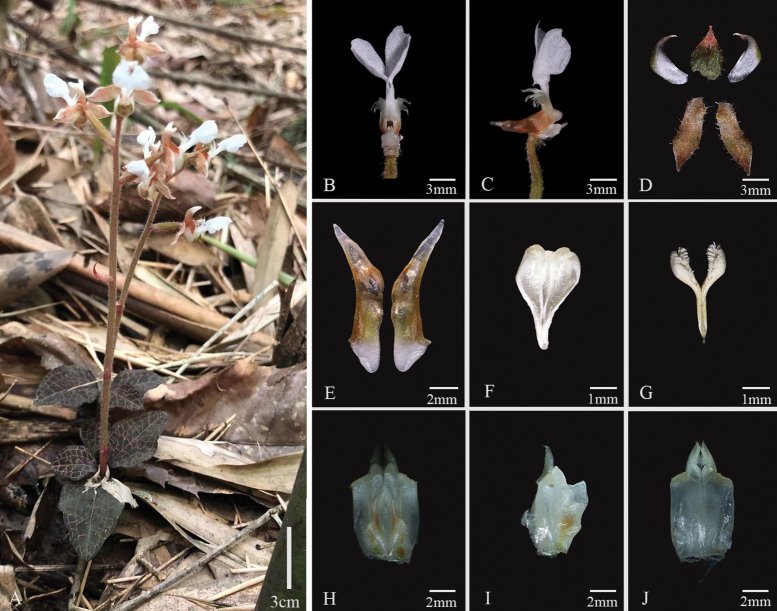
*Anoectochiluszhejiangensis***A** habit **B** flower (front view) without petals and sepals **C** flower (lateral view) without petals and sepals **D** sepals and petals **E** spur **F** anther cap **G** pollinia **H** column (front view) **I** column (lateral view) **J** core column (rear view). Photographs by Yan-Bin Wu.

**Figure 5.**
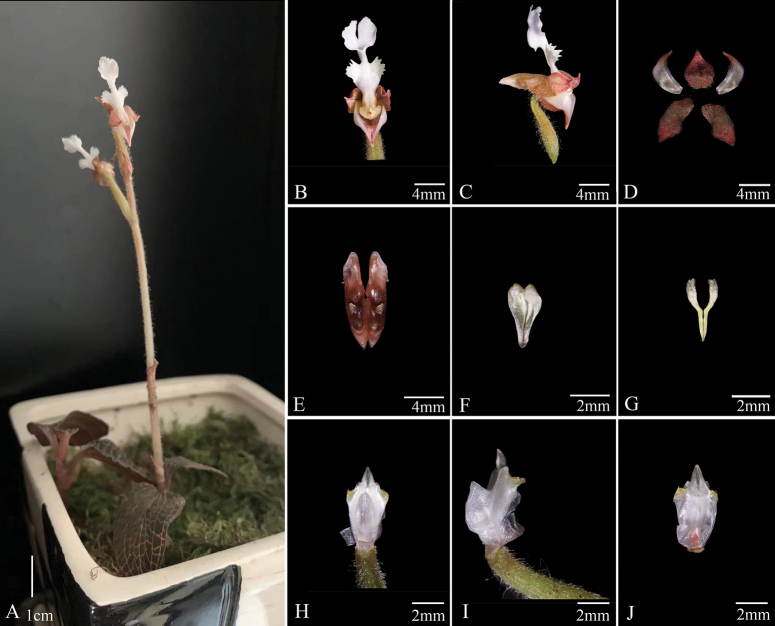
*Anoectochilusmalipoensis***A** transplant **B** flower (front view) **C** flower (lateral view) **D** sepals and petals **E** spur **F** anther cap **G** pollinia **H** column (front view) **I** column (lateral view) **J** core column (rear view). Photographs by Yan-Bin Wu.

*Anoectochiluszhongshanensis* (Fig. 3), collected from Guangxi, China, possesses typical features of *Anoectochilus* species, characterized by golden-vein foliage, conical spur, separated stigma-lobes, and the pair of lamellae on column. This new species shows close morphological similarity to *A.zhejiangensis* and *A.malipoensis* (Figs 4, 5), but can be distinguished by the hastate or scalpel-shaped lobes of epichile, forward curved and pinnately divided cristate lobes at both sides of the mesochile, and inverted triangle column wings. Molecular studies based on cpDNA (*rbcL*-*matK*-*trnl*-*F*) combined with chemical analysis on characteristic lactone glycoside further supported the uniqueness of this new species. The key for identifications of 20 already reported species of genus *Anoectochilus* in China has been recently and systematically established by Jin et al (Jin et al. 2021), and *A.zhongshanensis* is thus the 21^st^ member that can be distinguished from its nearest congeners, *A.zhejiangensis* and *A.malipoensis*.

The single use of nrDNA (ITS) (Suppl. materials 1–3) or concatenation of both nrDNA and cpDNA (ITS, *rbcL*, *matK*, *trnl*-*F*) (Suppl. material 5) failed to establish distinguishable relationships among those tested *Anoectochilus* species. However, only combined cpDNA resulted in a more resolved topology with a reliable phylogenetic result consistent with previous studies (Han 2019; Jin et al. 2021). These inconsistences can be explained by the varied evolution between nuclear ribosomal DNA and chloroplast DNA (Clegg et al. 1994; Pérez-Escobar et al. 2020), which makes nrDNA ITS much more suitable for genetic diversity analysis due to its faster evolutionary rate and cpDNA better for genetic relationship investigation in virtue of their prolific hereditary information. Based on a series of new species (Tian and Xing 2008; Chen and Shui 2010; Tian et al. 2014; Qu et al. 2015) and new records for China (Hu et al. 2012; Wu et al. 2017; Zheng et al. 2018; Jin et al. 2019; Zheng et al. 2019; Jin et al. 2021), there are currently 21 *Anoectochilus* species known from China. And more distinguishable DNA molecular markers are needed to be developed to provide convincing species delimitation in the *Anoectochilus* genus.

**Figure 6.**
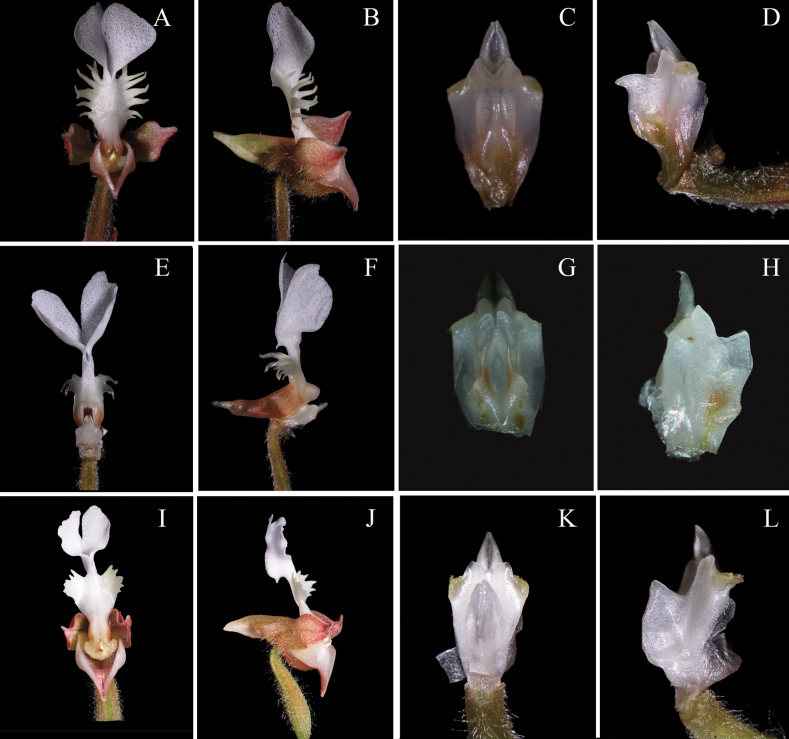
Diagnostic morphologic characteristics comparing *A.zhongshanensis* (**A–D**), *A.zhejiangensis* (**E–H**) and *A.malipoensis* (**I–L**) **A, E, I** flower (front view) **B, F, J** flower (lateral view) **C, G, K** column (front view) **D, H, L** column (lateral view). Photographs by Yan-Bin Wu.

**Figure 7.**
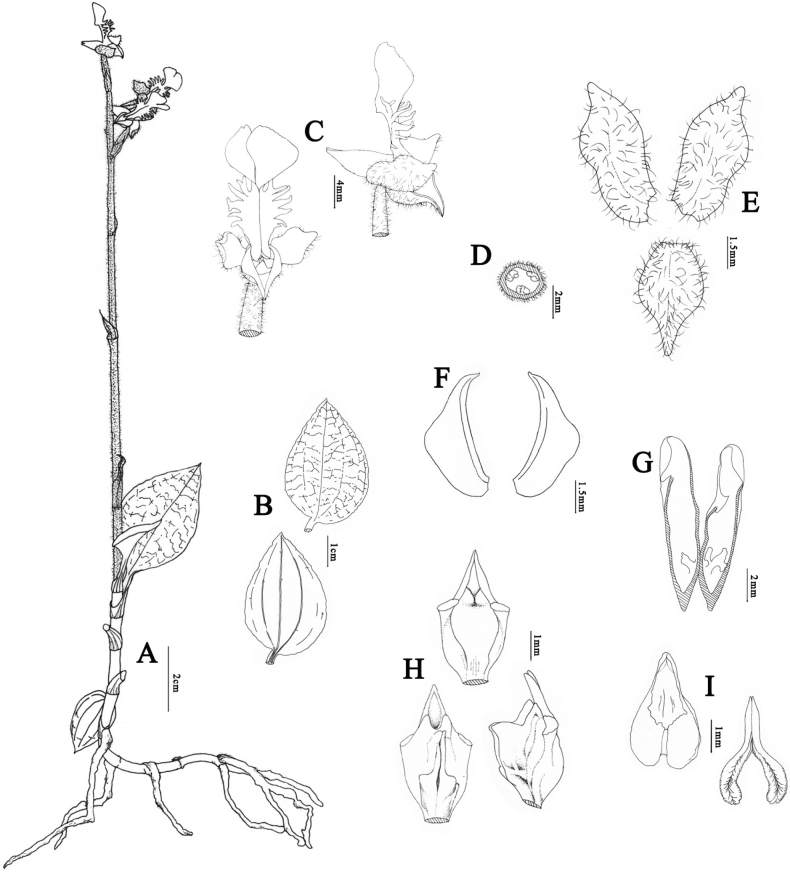
*Anoectochiluszhongshanensis***A** habit **B** leaves **C** flower (front view and lateral view) **D** ovary (cross section) **E** sepals **F** petals **G** spur **H** core column (front view, lateral view and rear view) **I** anther cap and pollinia. Drawn by Li-Xiang Zheng.

## Supplementary Material

XML Treatment for Anoectochilus zhongshanensis

## ﻿Additional information

### Author ORCIDs

Yan-Bin Wu https://orcid.org/0000-0001-9401-366X

Cheng-Jian Zheng https://orcid.org/0000-0002-7867-3438

### Data availability

All of the data that support the findings of this study are available in the main text or Supplementary Information.

## Supplementary materials

Supplementary material 1 Phylogenetic relationships based on nrDNA (ITS) in *Anoectochilus* species inferred by maximum likelihood (ML) Data type jpg https://binary.pensoft.net/file/926187This dataset is made available under the Open Database License (http://opendatacommons.org/licenses/odbl/1.0/). The Open Database License (ODbL) is a license agreement intended to allow users to freely share, modify, and use this Dataset while maintaining this same freedom for others, provided that the original source and author(s) are credited.Yan-Bin Wu, Yu Han, Xu-Hui He, Hui-Ling Chen, Jin-Zhong Wu, Qi Ye, Cheng-Jian Zheng

Supplementary material 2 Phylogenetic relationships based on nrDNA (ITS) in *Anoectochilus* species inferred by Bayesian inference (BI) Data type jpg https://binary.pensoft.net/file/926188This dataset is made available under the Open Database License (http://opendatacommons.org/licenses/odbl/1.0/). The Open Database License (ODbL) is a license agreement intended to allow users to freely share, modify, and use this Dataset while maintaining this same freedom for others, provided that the original source and author(s) are credited.Yan-Bin Wu, Yu Han, Xu-Hui He, Hui-Ling Chen, Jin-Zhong Wu, Qi Ye, Cheng-Jian Zheng

Supplementary material 3 Phylogenetic relationships based on nrDNA (ITS) in *Anoectochilus* species inferred by maximum parsimony (MP) Data type jpg https://binary.pensoft.net/file/926189This dataset is made available under the Open Database License (http://opendatacommons.org/licenses/odbl/1.0/). The Open Database License (ODbL) is a license agreement intended to allow users to freely share, modify, and use this Dataset while maintaining this same freedom for others, provided that the original source and author(s) are credited.Yan-Bin Wu, Yu Han, Xu-Hui He, Hui-Ling Chen, Jin-Zhong Wu, Qi Ye, Cheng-Jian Zheng

Supplementary material 4 Phylogenetic relationships based on concatenated *rbcL*, *matK* and *trnL*-*F* sequences in *Anoectochilus* species inferred by maximum parsimony (MP) Data type png Explanation note BSMP is shown below the brancher. https://binary.pensoft.net/file/926190This dataset is made available under the Open Database License (http://opendatacommons.org/licenses/odbl/1.0/). The Open Database License (ODbL) is a license agreement intended to allow users to freely share, modify, and use this Dataset while maintaining this same freedom for others, provided that the original source and author(s) are credited.Yan-Bin Wu, Yu Han, Xu-Hui He, Hui-Ling Chen, Jin-Zhong Wu, Qi Ye, Cheng-Jian Zheng

Supplementary material 5 Phylogenetic relationships based on concatenated ITS, *rbcL*, *matK* and *trnL*-*F* sequences in *Anoectochilus* species inferred by Bayesian inference (BI) Data type jpg https://binary.pensoft.net/file/926191This dataset is made available under the Open Database License (http://opendatacommons.org/licenses/odbl/1.0/). The Open Database License (ODbL) is a license agreement intended to allow users to freely share, modify, and use this Dataset while maintaining this same freedom for others, provided that the original source and author(s) are credited.Yan-Bin Wu, Yu Han, Xu-Hui He, Hui-Ling Chen, Jin-Zhong Wu, Qi Ye, Cheng-Jian Zheng

Supplementary material 6 Information of samples for phylogenetic analysis in this study Data type docx https://binary.pensoft.net/file/926192This dataset is made available under the Open Database License (http://opendatacommons.org/licenses/odbl/1.0/). The Open Database License (ODbL) is a license agreement intended to allow users to freely share, modify, and use this Dataset while maintaining this same freedom for others, provided that the original source and author(s) are credited.Yan-Bin Wu, Yu Han, Xu-Hui He, Hui-Ling Chen, Jin-Zhong Wu, Qi Ye, Cheng-Jian Zheng

Supplementary material 7 Primers and amplification protocols used in this study Data type docx https://binary.pensoft.net/file/926193This dataset is made available under the Open Database License (http://opendatacommons.org/licenses/odbl/1.0/). The Open Database License (ODbL) is a license agreement intended to allow users to freely share, modify, and use this Dataset while maintaining this same freedom for others, provided that the original source and author(s) are credited.Yan-Bin Wu, Yu Han, Xu-Hui He, Hui-Ling Chen, Jin-Zhong Wu, Qi Ye, Cheng-Jian Zheng
