# Supplementary figures and images for: ﻿Anoectochiluszhongshanensis (Orchidaceae), a new species from Guangxi, China
Source: PhytoKeys. 2023 Oct 25;234:203–18. doi: 10.3897/phytokeys.234.111106 (PMC10620710; doi:10.3897/phytokeys.234.111106)

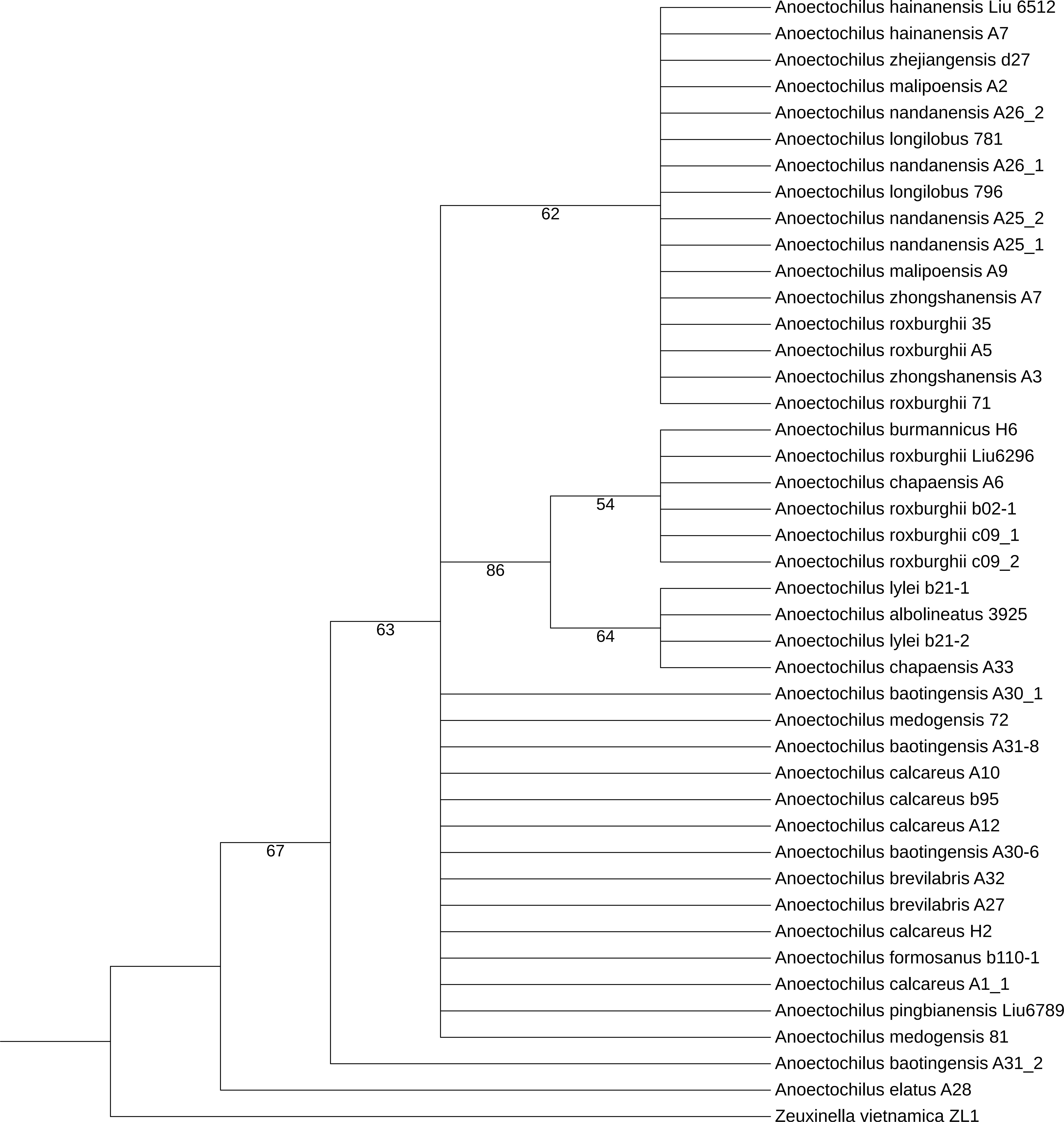

Supplement: Supplementary material 1 — Phylogenetic relationships based on nrDNA (ITS) in Anoectochilus species inferred by maximum likelihood (ML) [file phytokeys-234-203_article-111106__-s001.jpg]

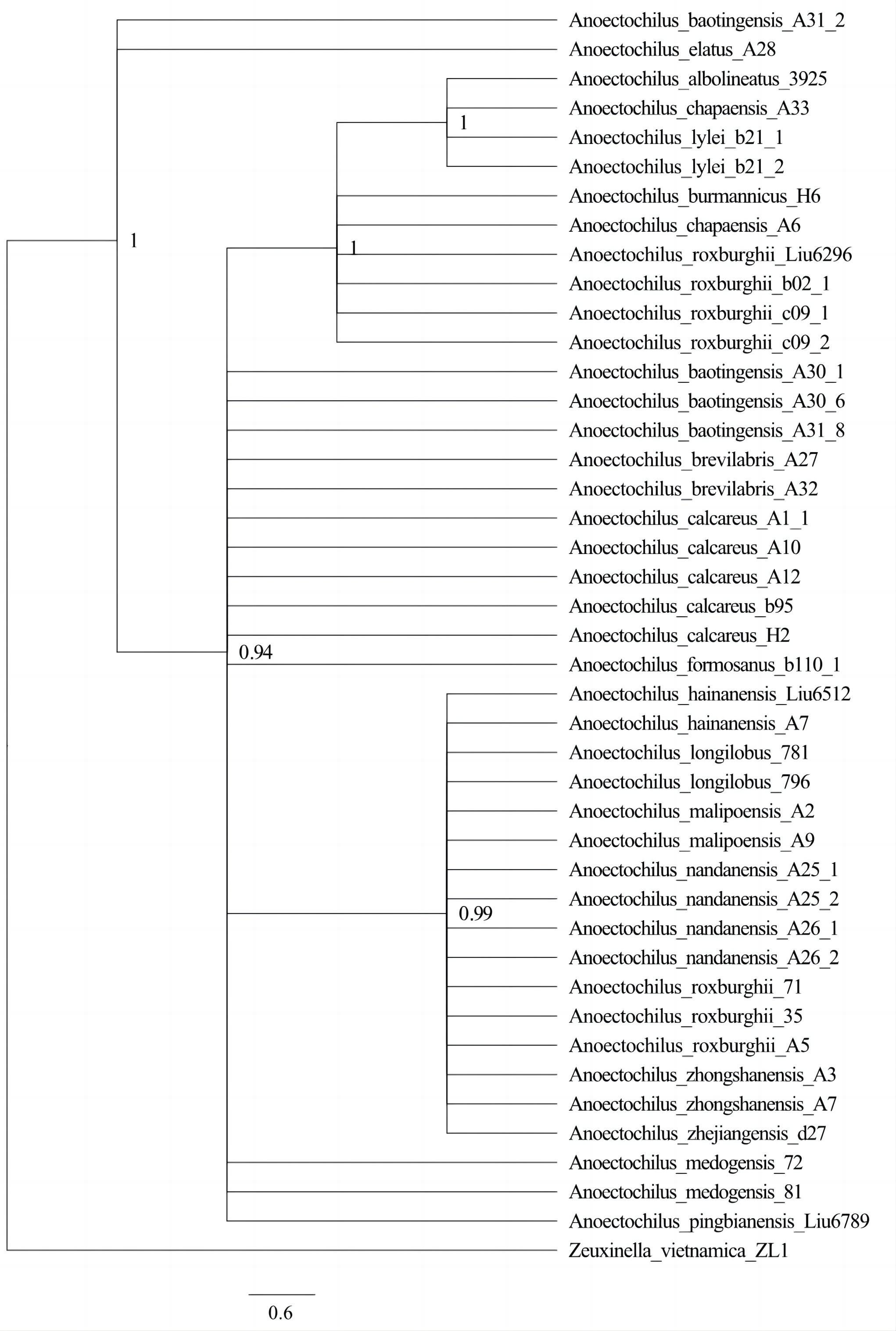

Supplement: Supplementary material 2 — Phylogenetic relationships based on nrDNA (ITS) in Anoectochilus species inferred by Bayesian inference (BI) [file phytokeys-234-203_article-111106__-s002.jpg]

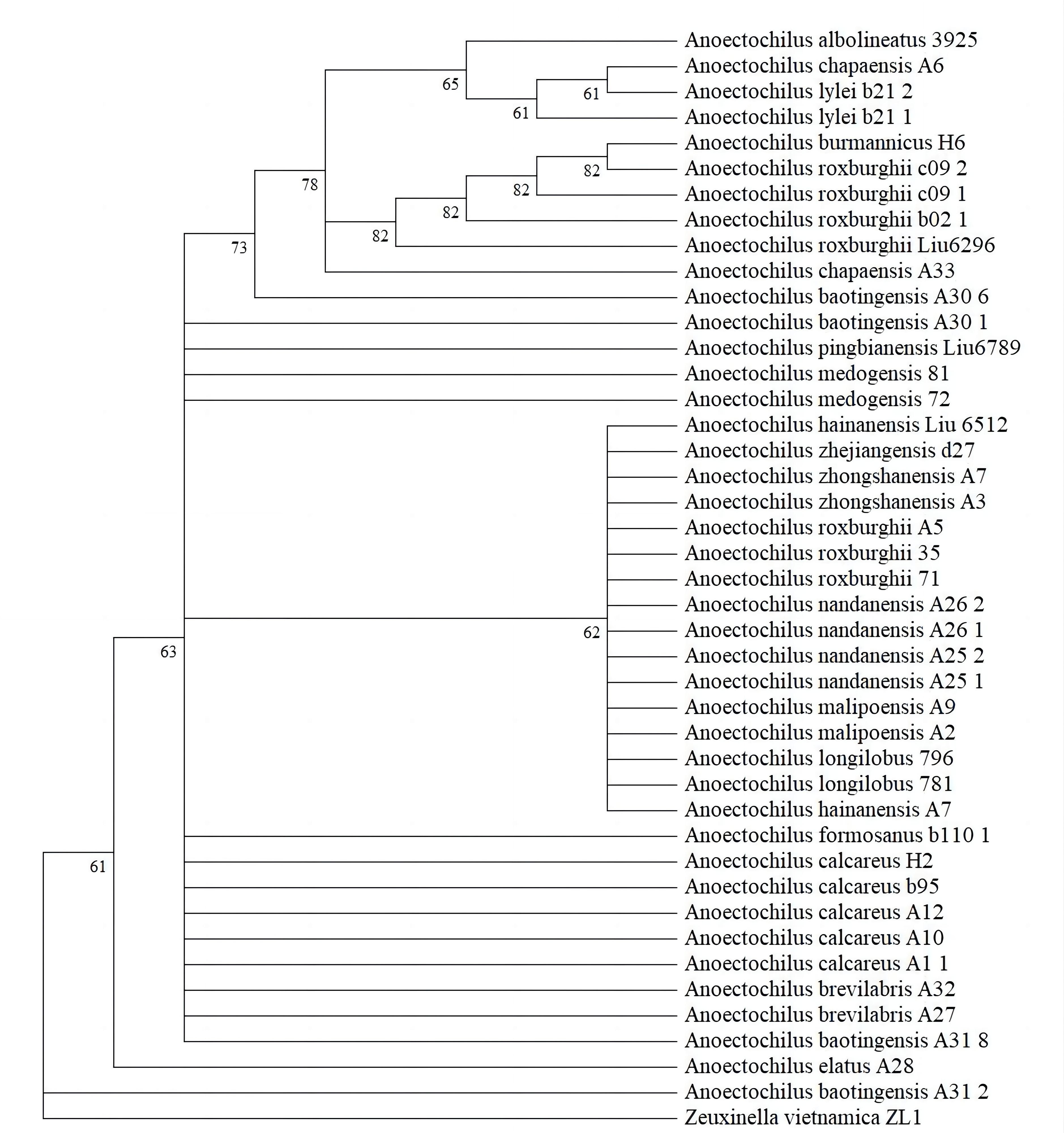

Supplement: Supplementary material 3 — Phylogenetic relationships based on nrDNA (ITS) in Anoectochilus species inferred by maximum parsimony (MP) [file phytokeys-234-203_article-111106__-s003.jpg]

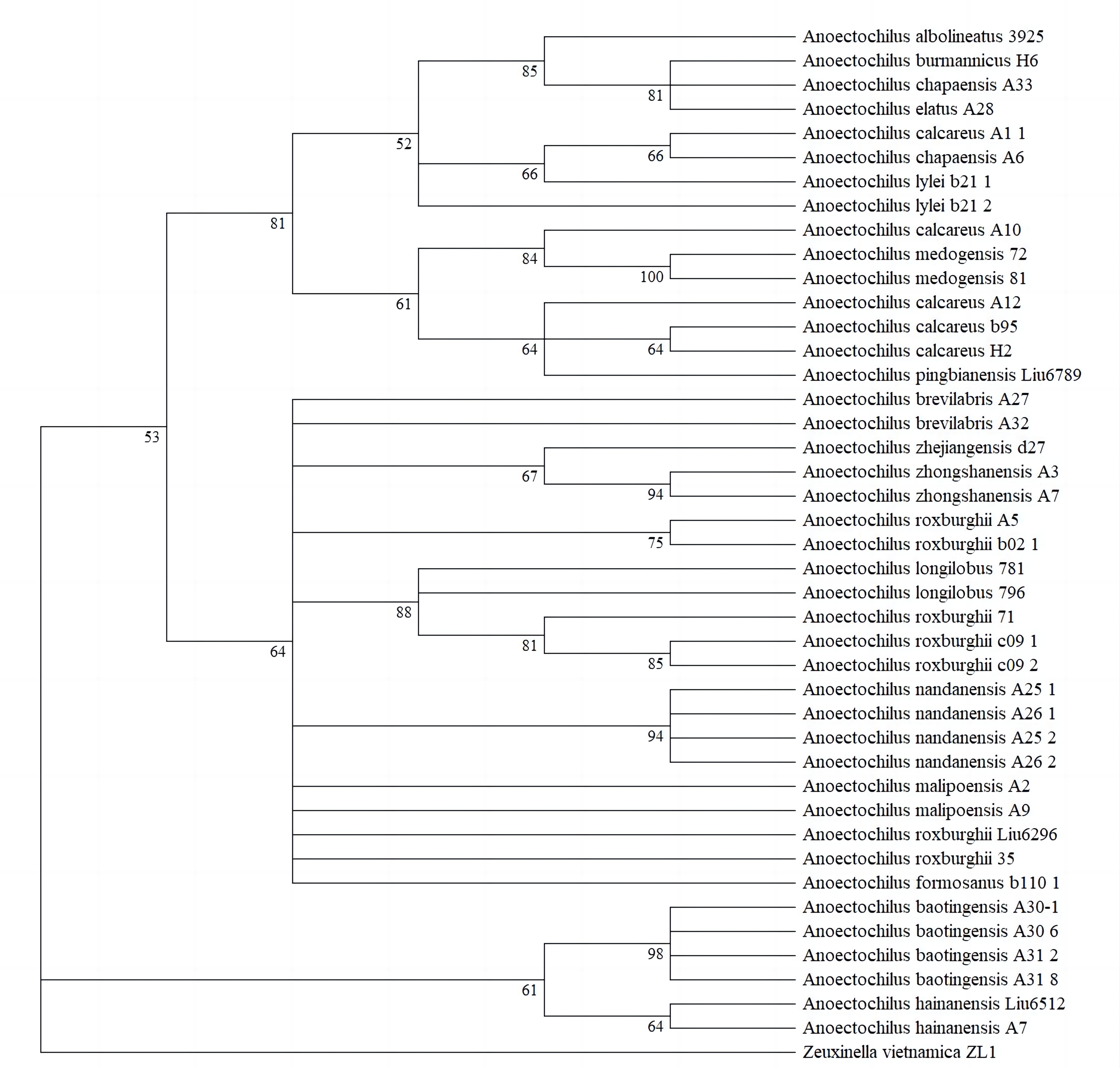

Supplement: Supplementary material 4 — Phylogenetic relationships based on concatenated rbcL, matK and trnL-F sequences in Anoectochilus species inferred by maximum parsimony (MP) [file phytokeys-234-203_article-111106__-s004.png]

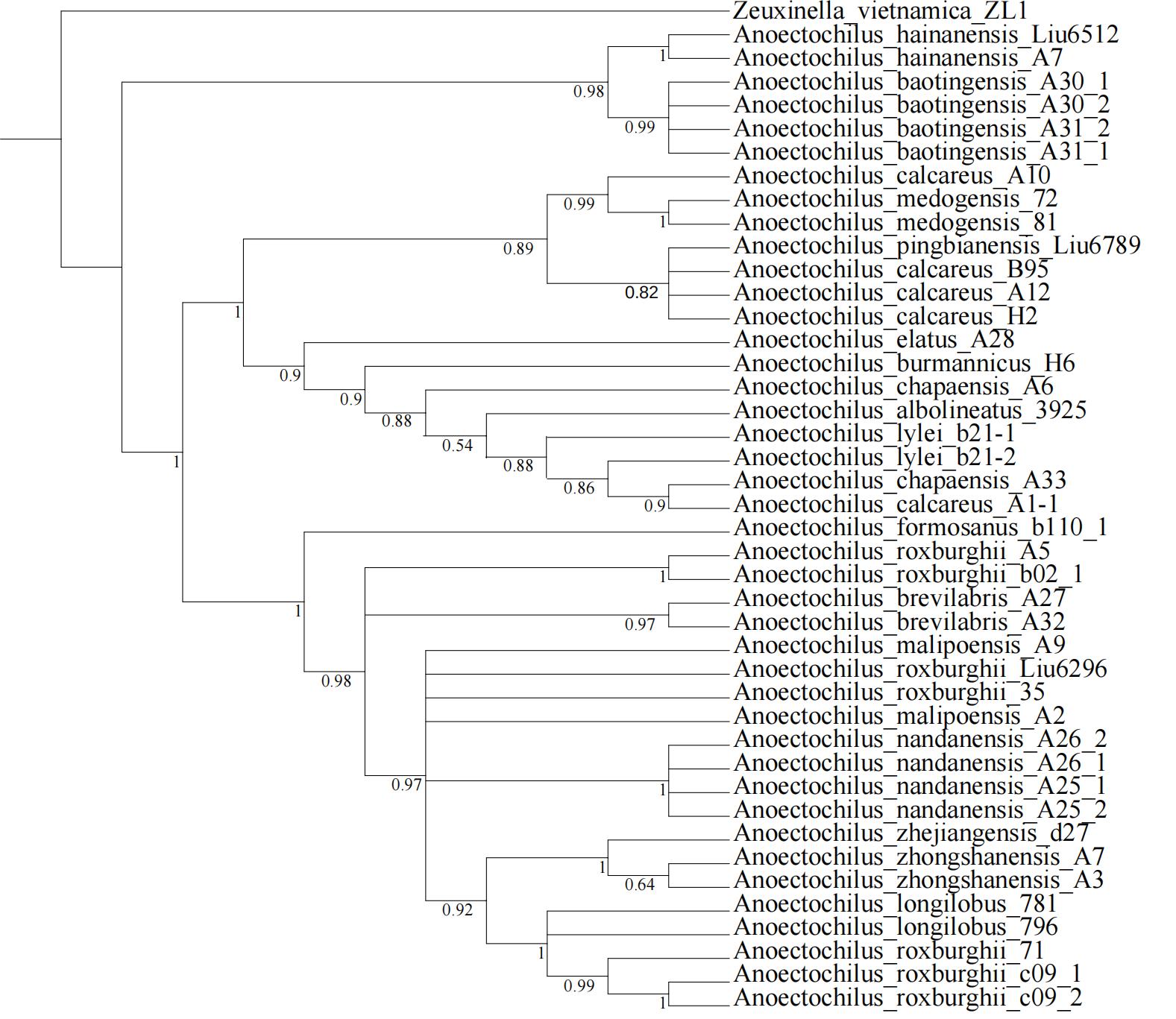

Supplement: Supplementary material 5 — Phylogenetic relationships based on concatenated ITS, rbcL, matK and trnL-F sequences in Anoectochilus species inferred by Bayesian inference (BI) [file phytokeys-234-203_article-111106__-s005.jpg]
