# Supplementary material for: ﻿Anoectochiluszhongshanensis (Orchidaceae), a new species from Guangxi, China
Source: PhytoKeys. 2023 Oct 25;234:203–18. doi: 10.3897/phytokeys.234.111106 (PMC10620710; doi:10.3897/phytokeys.234.111106)
Supplement: Supplementary material 6 — Information of samples for phylogenetic analysis in this study [file phytokeys-234-203_article-111106__-s006.docx]

**Table S1. Information of samples for phylogenetic analysis in this study.**

| Taxon | Locality | Date | Collector | Voucher | ITS | matK | trnL-F | rbcL |
| --- | --- | --- | --- | --- | --- | --- | --- | --- |
| *Anoectochilus albolineatus* | Jinghong,Xishuang banna,Yunnan | 2013.11.29 | Li Jianwu | 3925 | MW721044 | MW020662 | MW016893 | MW020621 |
| *Anoectochilus baotingensis 1* | Tongtieling,Wanni ng,Hainan | 2018.5.6 | Zheng Xilong | A30-1 | MW721045 | MW020663 | MW016894 | MW020622 |
| *Anoectochilus baotingensis 2* | Tongtieling,Wanni ng,Hainan | 2018.5.6 | Zheng Xilong | A30-6 | MW721046 | MW020664 | MW016895 | MW020623 |
| *Anoectochilus baotingensis 3* | Tongtieling,Wanni ng,Hainan | 2018.5.6 | Zheng Xilong | A31-2 | MW721047 | MW020665 | MW016896 | MW020624 |
| *Anoectochilus baotingensis 4* | Tongtieling,Wanni ng,Hainan | 2018.5.6 | Zheng Xilong | A31-8 | MW721048 | MW020666 | MW016897 | MW020625 |
| *Anoectochilus brevilabris 1* | Malipo,Yunnan | 2017.7.31 | Wu Yanbin | A27 | MW721049 | MW020698 | MW016929 | MW020657 |
| *Anoectochilus brevilabris 2* | Guangxi,Sino Vietnamese Border | 2018.6.4 | Wu Lei | A32 | MW721050 | MW020667 | MW016898 | MW020626 |
| *Anoectochilus burmannicus 1* | Jinghong,Xishuang banna,Yunnan | 2015.7.15 |  | H6 | MW721051 | MW020668 | MW016899 | MW020627 |
| *Anoectochilus chapaensis* | Mengla,Xishuangb anna,Yunnan | 2011.12 | Zhao Mingxu | A6 | MT872101 | MT887673 | MT872535 | MW020660 |
| *Anoectochilus chapaensis 1* | Lushui,Yunnan | 2018.7.31 | Jiang Hong | A33 | MW721056 | MW020673 | MW016904 | MW020632 |
| *Anoectochilus calcareus 1* | Xinjiacun,Guizhou | 2013.8.1 | Tian Huaizhen | A1-1 | MT872100 | MT887672 | MT872534 | MW020658 |
| *Anoectochilus calcareus 2* | Guangxi,Sino Vietnamese Border | 2013.8.6 | Huang Yunfeng | A10 | MW721052 | MW020669 | MW016900 | MW020628 |
| *Anoectochilus calcareus 3* | Nanning,Guangxi | 2014.8.28 | Lai Bidan | A12 | MW721053 | MW020670 | MW016901 | MW020629 |
| *Anoectochilus calcareus 4* | - | - | Wang Bingmou | b95 | MW721054 | MW020671 | MW016902 | MW020630 |
| *Anoectochilus calcareus 5* | Guangxi,Sino Vietnamese Border | 2014.8.28 | - | H2 | MW721055 | MW020672 | MW016903 | MW020631 |
| *Anoectochilus elatus* | Jinghong,Xishuang banna,Yunnan | 2016.12.3 |  | A28 | MW721057 | MW020674 | MW016905 | MW020633 |
| *Anoectochilus formosanus* | Taibei,Taiwan | 2011.12.28 | Tian Huaizhen | b110-1 | MW721058 | MW020675 | MW016906 | MW020634 |
| *Anoectochilus hainanensis 1* | - | - | - | Liu 6512 | MK451730 | MK451792 | MK451771 | MK451834 |
| *Anoectochilus hainanensis 2* | Hainan | 2013.12.4 | Huang Mingzhong | A7 | MT872102 | MT887674 | MT872536 | MW020661 |
| *Anoectochilus longilobus 1* | Pinbian,Yunnan | 2013.7.27 | Tian Huaizhen | 781 | MW721059 | MW020676 | MW016907 | MW020635 |
| *Anoectochilus longilobus 2* | Pinbian,Yunnan | 2013.7.27 | Tian Huaizhen | 796 | MW721060 | MW020677 | MW016908 | MW020636 |
| *Anoectochilus lylei 1* | Xishuangbanna, Yunnan | 2011.7.9 | Hu Chao | b21-1 | MT872103 | MT887675 | MT872537 | MW020659 |
| *Anoectochilus lylei 2* | Xishuangbanna, Yunnan | 2011.7.9 | Hu Chao | b21-2 | MW721061 | MW020678 | MW016909 | MW020637 |
| *Anoectochilus malipoensis 1* | Malipo,Yunnan | 2013.6.13 |  | A2 | MW721062 | MW020679 | MW016910 | MW020638 |
| *Anoectochilus malipoensis 2* | Guangxi | 2017.9.2 | Tian Huaizhen | A9 | MW721063 | MW020680 | MW016911 | MW020639 |
| *Anoectochilus medogensis 1* | Motuo,Xizang | 2019.8.17 | Tian Huaizhen | 72(20180817002) | MW721064 | MW020681 | MW016912 | MW020640 |
| *Anoectochilus medogensis 2* | Motuo,Xizang | 2019.8.17 | Tian Huaizhen | 81(20180818001) | MW721065 | MW020682 | MW016913 | MW020641 |
| *Anoectochilus nandanensis 1* | Longlin,Guangxi | 2014.8 | Li Dahong | A25-1 | MW721066 | MW020683 | MW016914 | MW020642 |
| *Anoectochilus nandanensis 2* | Longlin,Guangxi | 2014.8 | Li Dahong | A25-2 | MW721067 | MW020684 | MW016915 | MW020643 |
| *Anoectochilus nandanensis 3* | Guangxi | 2018.4.25 | Wang Bingmou | A26-1 | MW721068 | MW020685 | MW016916 | MW020644 |
| *Anoectochilus nandanensis 4* | Guangxi | 2018.4.25 | Wang Bingmou | A26-2 | MW721069 | MW020686 | MW016917 | MW020645 |
| *Anoectochilus pingbianensis* | - | - | - | Liu 6789 | MK451731 | MK451793 | MK451772 | MK451835 |
| *Anoectochilus roxburghii 1* | - | - | - | Liu 6296 | MK451732 | MK451794 | MK451773 | MK451836 |
| *Anoectochilus roxburghii 2* | Motuo,Xizang | 2019.8.17 | Tian Huaizhen | 71(20180817001) | MW721070 | MW020687 | MW016918 | MW020646 |
| *Anoectochilus roxburghii 3* | Daiyunshan,Fujian | 2013.10.1 | Cheng Zhiquan | 35 | MW721071 | MW020688 | MW016919 | MW020647 |
| *Anoectochilus roxburghii 4* | Damingshan, Guangxi | 2013.8.13 | Cheng Zhiquan | A5 | MW721072 | MW020689 | MW016920 | MW020648 |
| *Anoectochilus roxburghii 5* | Kaihua,Guangdong | 2011.4.8 | Hu Chao | b02-1 | MW721073 | MW020690 | MW016921 | MW020649 |
| *Anoectochilus roxburghii 6* | Yunnan | 2012.6-7 | Tong Yi | c09-1 | MW721075 | MW020692 | MW016923 | MW020651 |
| *Anoectochilus roxburghii 7* | Yunnan | 2012.6-7 | Tong Yi | c09-2 | MW721076 | MW020693 | MW016924 | MW020652 |
| *Z**euxinella vietnamica* | Guangxi | 2013.12.10 | Wang Bingmou | ZL1 | MW721079 | MW020697 | MW016928 | MW020656 |
| *A**noectochilus zhejiangensis* | Daiyunshan,Fujian | 2011.8.26 | Wang Youfang | d27 | MT872106 | MT887677 | MT872540 | MW020655 |
| *Anoectochilus zhongshanensis* | Guangxi | 2020.8.12 | Wu Yanbin | A3 | OQ888186 | OR045916 | OR039287 | OR039286 |
| *Anoectochilus zhongshanensis* | Guangxi | 2020.8.12 | Wu Yanbin | A7 | OQ888220 | OR045917 | OR039288 | OR039289 |
