# Supplementary material for: ﻿Anoectochiluszhongshanensis (Orchidaceae), a new species from Guangxi, China
Source: PhytoKeys. 2023 Oct 25;234:203–18. doi: 10.3897/phytokeys.234.111106 (PMC10620710; doi:10.3897/phytokeys.234.111106)
Supplement: Supplementary material 7 — Primers and amplification protocols used in this study [file phytokeys-234-203_article-111106__-s007.docx]

**Table S2. Primers and amplification protocols used in this study.**

| **Region** | **Sequence (5′→3′)** | **Amplification protocols** |
| --- | --- | --- |
| ITS | 18sdir: CGTAACAAGGTTTCCGTAGG  ITS4: TCCTCCGCTTATTGATATGC | 95℃ 5 min, 94℃ 30 s, 58℃ 30 s, 72℃ 30 s, 45 cycles, 72℃ 10 min |
| matk1 | 19F: CGTTCTCATATTGCACTATG  834R: AAAGACTCCARAAGATRTTG |  |
| matk2 | 731F: TCTGGAGTCTTTCTTGAGCGA  trnK-2R: AACTAGTCGGATGGAGTAG |  |
| rbcL | rbcL-F: GTTGTAGGGAGGGACTTATGT  rbcL-R: TAGTTCAGGGCTCCATTTG |  |
| trnL-F | Forward c: CGAAATCGGTAGACGCTACG  Reverse f: ATTTGAACTGGTGACACGAG |  |
